# Content of selected inorganic compounds in the eggs of hens kept in two different systems: organic and battery cage

**DOI:** 10.5194/aab-62-431-2019

**Published:** 2019-07-18

**Authors:** Edyta Szymanek, Katarzyna Andraszek, Dorota Banaszewska, Kamil Drabik, Justyna Batkowska

**Affiliations:** 1 Department of Animal Genetics and Horse Breeding, Siedlce University of Natural Sciences and Humanities, Siedlce, 08-110, Poland; 2 Department of Breeding Methods and Poultry Breeding, Siedlce University of Natural Sciences and Humanities, Siedlce, 08-110, Poland; 3 Institute of Biological Basis of Animal Production, University of Live Science in Lublin, Lublin, 20-950, Poland

## Abstract

Recent years have seen increased interest in the influence of bioactive dietary components on human genes and gene expression. A good source of many bioactive substances is the chicken egg. The egg is considered to be an excellent food provided by nature. It is a good source of nutrients such as vitamins A, B2, B6, B12, D, E and K, as well as elements including phosphorus, selenium, iron, zinc, magnesium and calcium. The research material use in this study consisted of eggs from hens kept in two different systems: organic and battery cages. The content of calcium (Ca), magnesium (Mg) and zinc (Zn) was determined in the egg contents – in the yolk and white respectively. The content of elements was determined by atomic absorption spectrometry (AAS) using an AA280 FS spectrometer with the automatic dilution of standards and samples. The eggs from the organically raised hens had a higher calcium, magnesium and zinc content. The greater variation in the Ca, Mg and Zn content in the organic eggs is due to the more individualized feeding system. The rearing system of the hens significantly affects the concentration of elements in the egg. The results of this research indicate that eggs from organic farming systems have a richer chemical composition in terms of the content of nutrients such as calcium, magnesium and zinc compared with eggs obtained from caged hens. Therefore, consumers purchasing eggs should consider the system in which the hens were reared, as eggs can be a valuable source of these elements in the diet.

## 1 Introduction

Until recently, the science of human nutrition was focused on nutrient deficiencies and their consequences. However, over the last decade or so, advancements have been made in our ability to study the molecular structure of an organism, which has enabled more thorough analysis of the mechanisms responsible for the proper functioning of the human body. There are currently many supporters of the theory that a sufficient knowledge of the human genome will make it possible to compose an individual diet for the body of a specific individual. There are two scientific disciplines dealing with this subject: nutrigenomics and nutrigenetics.

Nutrigenomics investigates the effect of the bioactive dietary components on gene expression (the genome, transcriptome, proteome and metabolome); analyses the relationship between diet and genetic predispositions for diseases of civilization, including cancer and metabolic and cardiovascular disease; and identifies mechanisms determining how food and nutrition affect health (Chadwick, 2004; Gętek et al., 2013). This has to do with individualized nutrition, because the body's reaction to a nutrient depends on the genotype specific for the individual, determined by the sequence of base pairs forming the genome (Ordovas, 2008). Hence, nutrigenomics involves studying the reactions that take place between the nutrients and bioactive compounds contained in food and individual genetic traits (Chen and Kong, 2005; Frazer et al., 2009; Rimbach and Minihone, 2009). An important aspect of research concerning the interactions between genetic factors and environmental factors associated with the impact of nutrition on genome function is epigenetic and epigenomic analysis (Jirtle and Skinner, 2007; Moss and Wallrath, 2007). Diet alone and in combination with other environmental factors can affect epigenetic processes, causing the activation of genes that are silenced, and in some cases can lead to the development of disease. In addition, deregulation of the work of some groups of genes may increase the risk of disease manifested in later life (Fenech, 2005; Fenech et al., 2005; Sharma et al., 2010).

In the future, it will be possible to use this information about the interaction between nutrients and genes for the individual assessment of the risks or benefits that may arise from the consumption of specific groups of products or the use of a specific type of diet (Adamska and Ostrowski, 2010; Fenech, 2008; Fenech et al., 2011).

Due to changes in the living conditions of human beings and advances in sciences dealing with human nutrition, our diet differs significantly from that of people living even a few decades ago. Despite changes and numerous trends in nutrition, the goal should be a balanced diet, considered to be optimal or simply healthy. This is simply a diet that meets nutritional requirements.

The opinion on the use of eggs in human nutrition has changed diametrically over decades. Until recently, this assessment was focused on the high cholesterol content in eggs and the health consequences of a high cholesterol level. It was then fashionable to limit the consumption of eggs or even completely eliminate them from the diet. Recent studies, however, indicate that cholesterol from eggs has no negative effect on the serum cholesterol level (Hu et al., 2001). The time has come to re-evaluate eggs and focus on their nutritional value, their functional substances and even their positive role in maintaining genome stability.

The egg is an important source of energy, protein and other nutrients for human beings, and their rational consumption stimulates metabolic functions in the body and increases resistance to disease. It is also an excellent source of vitamins and minerals, especially digestible phosphorus and iron (Nys et al., 2011). Eggs should be a part of the daily diet, especially health-promoting designer eggs, also known as functional eggs (Surai and Sparks, 2001; Trziszka et al., 2013). Eggs are considered nutraceuticals, i.e. natural foods which are health-promoting and therapeutic (Nain et al., 2012). Designer eggs may contain several times more biologically active nutrients than ordinary table eggs (Mc Namara, 2010; Trziszka et al., 2013; Walczak et al., 2016). The great advantage of eggs as a source of nutrients is their high bioavailability (Wellman-Labadie et al., 2007; Kijowski et al., 2013).

According to scientific research carried out in recent years, eggs reduce the risk of many diseases, support the overall condition of the body and act as a natural supplement in the case of a nutrient-poor diet (Kritchevsky and Kritchevsky, 2000; Seuss-Baum, 2007). An important asset of eggs is that the content of some individual constituents (minerals, cholesterol, fatty acids profile) can be easily modulated by adjusting the chicken feed (Hargis, 1988; Lewis et al., 2000; Brodacki et al., 2018). Modifications of the diet are also the reason for the wide variation in the content of nutrients and elements in the egg described in various scientific studies. The intensification of poultry production has unfortunately led to negative changes in egg structure. High laying rates may have an adverse effect on the parameters of the egg shell and contents (Premavalli and Viswanagthan, 2004). In Poland, there are native breeds of laying hens, which, unlike commercial breeding lines, are ideal for organic farming, and also produce meat and eggs with unique taste qualities (Cywa-Benko, 2002; Calik and Krawczyk, 2012). Thus, the aim of this study was to determine the content of selected factors influencing gene expression in eggs, depending on the farming system in which the hens are reared.

## 2 Material and methods

The study was carried out according to the guidelines of the III Local Ethics Committee on Animal Testing in Warsaw. The research material consisted of eggs from hens kept in two different systems: organic and battery cages. The organic eggs came from hens of the Green-legged Partridge breed. The second group comprised eggs from battery farming, purchased in a supermarket belonging to one of the local retail chains. Twenty eggs from each group were analysed. The content of calcium (Ca), magnesium (Mg) and zinc (Zn) was determined in the egg contents – in the yolk and white respectively. In addition, the content of these elements was calculated in a homogeneous mixture based on the percentage shares of yolk and protein in the egg.

The content of elements was determined by atomic absorption spectrometry (AAS) using an AA280 FS spectrometer (Varian, Australia) with the automatic dilution of standards and samples. The measurement was based on the absorption of monochromatic electromagnetic radiation passing through the flame with free atoms of the element to be determined. Varian single-element hollow-cathode lamps (HCL) were used. A procedure was used to optimize the conditions for the determination of each element. The sample in the form of a solution was introduced into the air-acetylene flame. Under these conditions, the sample was atomized – the compounds were broken up into individual atoms, which absorbed a portion of the light passing through the flame (with a length characteristic for a given element). The content of the element in the sample was determined based on the amount of light that reached the detector – the photomultiplier. To prepare the sample for the analysis of the above-mentioned elements, a 0.5 g weighted sample of egg white and yolk was mineralized using 10 mL of ultra-pure nitric acid (HNO3) (Merck, Germany) using a MARSXpress microwave mineralizer (CEM, USA). This process was carried out in three stages, at a temperature of 90, 110 and 210 ${}^{\circ }$C and a power of 400, 800 and 1600 W. Based on the content of calcium, magnesium and zinc determined in the yolk and albumen, the content of these elements in the total egg contents was calculated. The mean percentage share of egg yolk and albumen were used for the calculations.

The influence of the farming system on the content of elements was assessed by one-way analysis of variance using the following mathematical model:

1 Yij=μ+ai+eij,

where Yij is the value of the trait tested, μ is the mean for the population, ai is the effect of i-th level of factor (farming system) and eij is the sampling error.

Data were analysed by ANOVA using STATISTICA PL (2011) 10.0 software (STATISTICA version 10.0, StatSoft Inc., PL). The mean values (x) and the SD of measured traits were calculated, and the significant differences were evaluated using an F test. The level of significance was set at P≤0.05.

## 3 Results

The content of Ca, Mg and Zn in the yolk of eggs from the battery-cage and organic systems differed significantly at P≤0.05. Higher concentrations of each of the elements tested were found in the egg yolk of hens raised in the organic system; in the case of calcium these values were only slightly higher, but in the case of magnesium and zinc they were about twice as high. The system in which the hens were reared affected the content of calcium, magnesium and zinc in the egg yolk (Table 1).

**Table 1:** Content of Ca, Mg and Zn in egg yolk depending on the farming system.

Elements in	Farming system		
egg yolk	Battery cages	Organic	Total
	Mean ± SD	Mean ± SD	Mean ± SD
Ca (mg 100 g${}^{-1}$)	$127.38^{a}\pm 1.97$	$150.19^{b}\pm 4.51$	138.79±12.04
Mg (mg 100 g${}^{-1}$)	$14.84^{a}\pm 0.79$	$28.97^{b}\pm 2.12$	21.90±7.32
Zn (mg 100 g${}^{-1}$)	$2.97^{a}\pm 0.09$	$5.02^{b}\pm 0.38$	4.00±1.07

Mean values in rows marked with different letters ${}^{a}$ and ${}^{b}$ differ at P≤0.05.

The content of the analysed elements in the albumen of eggs from the battery-cage and organic systems differed significantly at P≤0.05. A significantly lower content of these elements was observed in the albumen than in the yolk. However, in the case of the albumen the average content of Ca, Mg and Zn was higher in the eggs from the organic farming system than the cage system. In the case of calcium and magnesium the differences were minor, but the concentration of zinc was three times as high. The system in which the hens were kept was found to affect the content of calcium, magnesium and zinc in the egg white (Table 2).

**Table 2:** Content of Ca, Mg and Zn in egg white depending on the farming system.

Elements in	Farming system		
egg white	Battery cages	Organic	Total
	Mean ± SD	Mean ± SD	Mean ± SD
Ca (mg 100 g${}^{-1}$)	$4.90^{a}\pm 0.13$	$5.27^{b}\pm 0.40$	5.09±0.35
Mg (mg 100 g${}^{-1}$)	$6.02^{a}\pm 0.41$	$8.23^{b}\pm 0.68$	7.13±1.24
Zn (mg 100 g${}^{-1}$)	$0.19^{a}\pm 0.01$	$0.60^{b}\pm 0.04$	0.40±0.20

Mean values in rows marked with different letters ${}^{a}$ and ${}^{b}$ differ at P≤0.05.

In the mixture of both egg fractions, the same relationship was observed as in the previous cases: the average calcium, magnesium and zinc content was higher in eggs from organically raised chickens. Furthermore, as in the case of the yolk and albumen, the differences were statistically significant (P≤0.05). The greatest variation was found in the case of zinc, and the smallest for calcium. The farming system affected the calcium, magnesium and zinc content in the mixture of yolk and albumen (Table 3).

Table 4 presents aggregate results of the analyses of the content of the tested elements and how much higher the content of these elements was in organic eggs than in eggs from the battery-cage system.. The levels of Ca, Mg and Zn were higher in each of the fractions studied in eggs from the organic system. Higher variation (V%) in the content of the elements was also found in the eggs from the organic system compared with the cage system.

**Table 3:** Content of Ca, Mg and Zn in the mixture of egg yolk and white depending on the farming system.

Elements in white	Farming system		
and yolk mixture	Battery cages	Organic	Total
	Mean ± SD	Mean ± SD	Mean ± SD
Ca (mg 100 g${}^{-1}$)	$47.77^{a}\pm 0.67$	$55.99^{b}\pm 1.64$	51.88±4.34
Mg (mg 100 g${}^{-1}$)	$9.11^{a}\pm 0.35$	$15.49^{b}\pm 0.83$	12.30±3.29
Zn (mg 100 g${}^{-1}$)	$1.17^{a}\pm 0.03$	$2.15^{b}\pm 0.14$	1.66±0.50

Mean values in rows marked with different letters a and b differ at P≤0.05.

**Table 4:** Content of Ca, Mg and Zn in the yolk, white, and mixture of yolk and white depending on the farming system, and difference (in %) in the content of Ca, Mg and Zn in organic and battery-cage eggs.

Farming	Statistic	Yolk			White			Mixture		
system		Ca	Mg	Zn	Ca	Mg	Zn	Ca	Mg	Zn
	Mean	127.38	14.84	2.97	4.90	6.02	0.19	47.77	9.11	1.17
Cages	SD	1.97	0.79	0.09	0.13	0.41	0.01	0.67	0.35	0.03
	V%	1.55	5.36	3.30	2.79	6.83	6.34	1.42	3.94	2.98
	Mean	150.19	28.97	5.02	5.27	8.23	0.60	55.99	15.49	2.15
Organic	SD	4.51	2.12	0.38	0.40	0.68	0.04	1.64	0.83	0.14
	V%	3.00	7.32	7.67	7.67	8.27	7.14	2.94	5.40	6.95
Organic/Cage		18 %	95 %	69 %	8 %	37 %	216 %	17 %	70 %	84 %

Organically farmed eggs had higher content of the elements studied in the yolk, white, and the mixture of yolk and white. The smallest variation in the content of the elements was noted for calcium, where the level in organic eggs was from 8 % (albumen) to 18 % (yolk) higher than in cage eggs. The greatest differences were found in the case of zinc. In organic eggs, the Zn content in the albumen was more than 200 % higher than in eggs from the cage system. In addition, the content of this element in the yolk and the mixture was over 50 % higher than in the corresponding components of cage eggs. It is also worth noting the high (over 90 % higher) content of Mg in the yolk of organic eggs.

## 4 Discussion

The problem of hunger and malnutrition is currently understood not only as a shortage of food, but also as a limited content of nutrients. The most commonly discussed nutrients are minerals, for which the role in maintaining homeostasis in the body is fairly well understood. The most important minerals in human nutrition include calcium, potassium, magnesium and zinc.

The level of calcium in the egg yolk was found to be dependent on the rearing system. This may indicate the effect of feed on the concentration of this element, which has also been observed in the work of Brodacki et al. (2018). Interestingly, a report by Naber (1979) indicates that the level of calcium in the egg is difficult to modify through the diet of birds. The amount of calcium in the egg, irrespective of the rearing system, shows that eggs can supply minerals as well as protein. This is even more important, because calcium plays an important role in nervous system function by stimulating the release of neurotransmitters (Smith and Augustine, 1988) or by transferring nerve impulses. Studies indicate that calcium deficiencies remain a serious nutritional problem (Kumssa et al., 2015). Szablewski et al. (2013) analysed the content of selected elements in the edible parts of whole eggs of hens of four breeds included in a genetic resources conservation programme. They obtained the following results for calcium: Sussex, 44.2 mg 100 g${}^{-1}$; Rhode Island Red, 58.9 mg 100 g${}^{-1}$; Green-legged Partridge, 55.9 mg 100 g${}^{-1}$; and Yellow-legged Partridge 61.8 mg 100 g${}^{-1}$. The Ca content in the eggs of Green-legged Partridge hens in our study was very similar (55.99 mg 100 g${}^{-1}$). The results of the present study were compared with those reported by Bologa et al. (2013), who determined the content of elements in the yolk, white and a mixture of these components obtained from two farming systems: conventional (caged) and organic. They found a higher content of calcium in the albumen of eggs from the cage system. This was not confirmed by our research, in which the level of calcium was higher in every component of the organic eggs.

Magnesium deficiency has recently become common, and the number of available supplementation methods is continually growing. A fundamental problem in eliminating deficiencies of this element is its low degree of retention. Higher bioavailability of organic forms of magnesium has been demonstrated (Coudray et al., 2005). For this reason it is worth noting the results of the present study, indicating its content in individual parts of the egg. Research by Kilić et al. (2002) demonstrated a relationship between the rearing system of birds and the amount of magnesium in individual morphological elements: the magnesium content was higher in both the yolk and the albumen of “country eggs” than in farm eggs. This tendency was statistically confirmed in the present study. This information is highly significant, as studies on the effects of magnesium deficiency indicate disturbances at the cell level, such as abnormalities in mitochondrial function or oxidative phosphorylation processes (Heaton and Rayssiguer, 1987), but they can also negatively affect the body more generally by causing anaemia (Tongyai et al., 1989) or myopathies (Riggs et al., 1992). As in the case of calcium, Bologa et al. (2013) reported a higher Mg content in the albumen and yolk-and-albumen mixture of conventional eggs than organic eggs. Heflin et al. (2018) demonstrated the influence of hen genotype (breed or hybrid type) on Mg content. In the same study, the level of this element was higher in eggs from the conventional rearing system and decreased with the intensity of the system.

The micronutrients analysed in this study have a significant influence on a number of regulatory processes; hence, it is worth discussing the possibility of supplying micronutrients using table eggs. Our research, similarly to that presented by Giannenas et al. (2009), indicates that eggs may be a valuable source of zinc. Interestingly, in the case of the yolk, these authors noted the opposite tendency to our observations, which indicate higher zinc concentrations in eggs from organic farms than from the conventional system. Our research did not confirm the results obtained by Bologa et al. (2013), in which the zinc level was higher in every component of conventional eggs. Heflin et al. (2018) found that this trait was influenced by the age of hens, but not by their genotype or the rearing system. Eggs from older hens had lower zinc content than eggs from younger hens. Relatively small differences in zinc content between breeds of hens have been reported by Szablewski et al. (2013). In addition, the value for Green-legged Partridge in our research was 2.15 mg 100 g${}^{-1}$, which was similar to the values reported in the cited paper.

Bioactive components of the egg can affect gene expression and genome stability. Calcium affects the stability of chromosome structure and prevents chromatid breaks; magnesium is involved in DNA replication and repair mechanisms; zinc is a cofactor of antioxidant enzymes; niacin contributes to telomere stability; choline prevents DNA damage and has an active role in regulating genome methylation; and vitamins C and E act as oxidants, including within nucleotides (Dreosti, 2001; Konopacka, 2004; Adamska and Ostrowski, 2010). All of these substances are found in the egg and are easily and well absorbed from it by the human body.

## 5 Conclusions

The results of the research indicate that eggs from organic farming have a richer chemical composition in terms of the content of nutrients such as calcium, magnesium and zinc compared with eggs obtained from caged hens. Therefore, consumers purchasing eggs should consider the system in which the hens were reared, as eggs can be a valuable source of these elements in the diet.

## Data Availability

Data availability. The data are available from the corresponding author upon request.
